# Introducing a group antenatal care model at the health post level in Ethiopia: a qualitative pilot study

**DOI:** 10.1186/s40814-026-01864-0

**Published:** 2026-06-17

**Authors:** Solomon Getachew Alem, Walelegn Worku Yallew, Della Berhanu, Konjit Wolde, Dedefo Teshite, Pooja Sripad, Lisa Noguchi, Stephanie Suhowatsky, Anne Hyre, Alemayehu Worku

**Affiliations:** 1 Addis Continental Institute of Public Health, Addis Ababa, Ethiopia; 2 Jhpiego Ethiopia, Addis Ababa, Ethiopia; 3 Department of Global Program and Technical Excellence, Jhpiego, Baltimore, MD 21231 USA; 4 School of Public Health, Addis Ababa University, Addis Ababa, Ethiopia

**Keywords:** Health post, Antenatal care, Group antenatal care, Health extension worker, Ethiopia, Rural, Pilot

## Abstract

**Background:**

Group antenatal care (G-ANC) is an alternative model of antenatal care that incorporates clinical assessment and care, participatory learning, and peer support. It gives expectant mothers the opportunity to discuss and share experiences with a group of women of similar gestational age throughout the pregnancy. This pilot study aimed to explore the feasibility of introducing a six-meeting G-ANC model in health posts, the most basic level of the health system in Ethiopia.

**Methods:**

A qualitative study of G-ANC was conducted at five purposively selected health posts in two zones in the Amhara region of Ethiopia, involving 54 pregnant women aged 15 years and older with a gestational age of less than 20 weeks. Trained research staff moderated 13 in-depth and 7 key informant interviews that were conducted after the second of the six G-ANC meetings. All interviews and discussions were recorded with consent from participants. Transcribed interviews were translated and thematically analyzed using a framework with open code qualitative data analysis software aiding in data sorting, categorization, and coding.

**Results:**

An adequate number of pregnant women were identified early in pregnancy and recruited to form G-ANC groups for this study. Pregnant women were willing to be enrolled and participate in G-ANC meetings and indicated interest in attending future meetings. Health extension workers (HEWs) demonstrated the ability to facilitate G-ANC meetings effectively.

**Conclusion:**

Introduction of G-ANC at the health post level appears feasible. None of the major anticipated feasibility challenges, adequate enrollment of eligible women, women’s willingness to participate in successive meetings, and HEW capacity to facilitate, were encountered. Existing community structures were instrumental in achieving adequate enrollment. The training and G-ANC meeting guides enabled HEWs to effectively facilitate the meetings. Preliminary results from this pilot study informed the design of a stepped-wedge cluster randomized trial to evaluate the acceptability and effectiveness of G-ANC at the health post level on increased coverage of antenatal care and facility-based delivery.

**Supplementary Information:**

The online version contains supplementary material available at 10.1186/s40814-026-01864-0.

## Key messages

- There was a lack of evidence on the feasibility of the G-ANC model at health post level. Despite successful implementation in higher-level and higher volume facilities in other countries, it remains unclear how this model would perform in Ethiopia’s most basic level of the health system, the health post.
- This study confirmed the feasibility of introducing G-ANC at health post level. An adequate number of eligible pregnant women of similar gestational age were identified and enrolled to form groups. Pregnant women were willing to participate in future G-ANC meetings. Health extension workers were effective in facilitating the G-ANC meetings.
- This pilot study informed the design of a cluster randomized stepped- wedged trial investigating the acceptability and effectiveness of G-ANC at the health post level.

## Background

Increased antenatal care (ANC) coverage is a crucial public health strategy for improving maternal and newborn health [1]. ANC provides critical interventions and counseling to promote positive birth outcomes[1]. Despite the benefits, many countries including Ethiopia still have low coverage of ANC, particularly four or more ANC [2]. ANC coverage in Ethiopia increased since 2005, but as of 2019 only 43% of pregnant women had four or more ANC visits [3–6]. Similarly, about half (48%) of women gave birth in a health facility [6]. Rural areas, compared to urban areas, have lower ANC coverage [7], higher home birth rates and higher maternal mortality [7, 8]. Several reasons contribute to the low ANC coverage including distance to facilities, long waiting times, lack of information about the importance of antenatal visits, and socio-cultural factors that discourage health-seeking behavior [9–11].

The Ministry of Health (MOH) of Ethiopia seeks innovative strategies to increase service utilization during pregnancy and childbirth in rural areas [12]. Group antenatal care (G-ANC) is an alternative service delivery model, compared to the individual ANC[1]. Women at their first ANC visit can opt for subsequent care in a group with 8–12 women of similar gestational age [8, 9]. The model incorporates medical assessment, knowledge sharing, and social support for pregnant women [13]. In G-ANC, each cohort participates in a series of 90–120-min meetings facilitated by a healthcare provider at the health facility [14]. This women-centered model aims to improve women’s access, engagement, and satisfaction with care, thus providing high quality ANC [11, 12]. Studies in high-income countries show G-ANC has resulted in reduced sexually transmitted infections (STIs); improved health literacy and patient satisfaction; increased ANC attendance and family planning uptake; reduced preterm births; and increased birth weights [15–19]. Several studies conducted in low and middle-income countries show G-ANC was associated with higher quality of ANC care, increased number of ANC contacts, and earlier postnatal care [20–23]. One study, conducted in a setting with a facility birth prevalence similar to Ethiopia, reported increased facility-based delivery in the intervention arm, compared to control [21].

In Ethiopia, the majority of health care services are provided at the primary level. The health post is the lowest level of the primary healthcare system, which primarily delivers promotive and preventive healthcare services, including ANC. One health post serves a population of 3000 to 5000 individuals within an administrative unit called a *kebele*, the smallest administrative unit [24]. Each health post is staffed by at least two health extension workers (HEWs). HEWs are salaried female community health workers, 18 years or older, recruited locally with knowledge of the local language. The HEWs in rural areas hold a minimum of a grade 10 education, compared to those in urban areas with a clinical nursing diploma [25, 26]. Health centers are the referral facilities for health posts and provide comprehensive maternal health services. Each health center is typically linked with approximately five health posts [24, 27, 28].

Under the standard individual ANC model at health posts, HEWs conduct focused antenatal assessments that include measurement of blood pressure, weight, and fundal height; identification of danger signs; provision of health education (e.g., nutrition, birth preparedness, danger sign recognition); distribution of iron-folate supplements and intermittent preventive treatment for malaria; and counseling on birth planning[29]. Point-of-care diagnostic testing for conditions such as anemia and syphilis has more recently been introduced at selected health posts through special projects[30]. Women requiring more advanced clinical services including ultrasound, comprehensive laboratory testing, or management of obstetric complications, are referred from health posts to the linked health center. Referral is facilitated by HEWs who accompany or communicate with health center staff to ensure continuity of care. This community-based model positions HEWs as primary ANC providers within the community, making them natural candidates to facilitate G-ANC in the same setting.

Across sub-Saharan Africa, G-ANC is primarily conducted at health centers and similar level facilities, with meetings facilitated by nurses and midwives [22, 31]. Less is known about where G-ANC can be implemented successfully at lower level facility such as the health posts. This pilot study served as a precursor to the main research on the acceptability and effectiveness of G-ANC in increasing antenatal care coverage and facility delivery at the health post level in Ethiopia. The pilot study aimed to explore the feasibility of introducing G-ANC facilitated by Health Extension Workers (HEWs). It assessed the availability of eligible pregnant women with similar gestational ages to form a G-ANC group; the willingness of these women to enroll and participate in future meetings; and the capacity of HEWs to facilitate the G-ANC meetings effectively.

## Methods and materials

### Study design

This pilot study was exploratory and used qualitative methods, mainly through in-depth and key informant interviews. This study informed a trial to evaluate the acceptability and effectiveness of the G-ANC implementation at the health post level utilizing a cluster randomized stepped-wedge study design.

### Study setting

The pilot study was conducted in two zones of Amhara region: West Gojjam zone in North Mecha district and South Gondar zone in Dera district. G-ANC meetings took place in 2021 at five purposively selected health posts under the catchment of two health centers in these two zones. This pilot study was conducted during the COVID-19 pandemic.

### Intervention

The Jhpiego’s G-ANC intervention model [20] initially designed for health centers was modified for the Ethiopian health post level in collaboration with the MOH. The aim was to enroll at least 10 pregnant women who were around 4–5 months pregnant from a health post catchment area to form a group. Women were invited to six G-ANC meetings, conducted monthly. Because health posts do not have full laboratory capacity for screening during ANC, point of care testing (POCT) also was implemented with support from the Enhancing Nutrition and Antenatal infection Treatment (ENAT) project, which was operating in the same region [32]. The POCT package included an asymptomatic bacteriuria dipstick test, hemoglobin level assessment, pregnancy urine dipstick test, and syphilis antibody rapid test. Ten HEWs, two from each of the five health posts, were trained for 5 days by Jhpiego staff on essential aspects of G-ANC implementation.

The pilot study was conducted during the first two of six G-ANC meetings in all five health posts. Each G-ANC meeting ran 90–120 min and followed a consistent structure involving peer-to-peer client self-assessment supervised by a HEW (e.g., weight and blood pressure measurement); private individual assessments by the HEW; highly participatory group discussions guided by pre-tested pictorial cards designed for non-literate audiences; plan and reflect sessions; and time for socially distanced socialization. Each meeting was documented as ANC contact. Meeting 1 focused on introducing group care and preventing pregnancy problems. Meeting 2 covered recognizing danger signs during pregnancy. Meetings were facilitated with a set of standardized materials, translated into Amharic.

Private clinical consultations were conducted by HEWs during or after meetings, and participants were encouraged to return at any time if they had questions or concerns. A simple register was used to collect G-ANC attendance data. Participants were reminded about the upcoming G-ANC meetings by phone calls from the HEWs when accessible and through peer-to-peer reminders. COVID-19 preventive measures were enforced during G-ANC meetings.

### Study participants and recruitment

Eligible study participants were: pregnant women aged 15 years and above who were less than 20 weeks of gestation at enrollment into G-ANC; HEWs who were trained to facilitate G-ANC meetings; and field staff who introduced the intervention (Jhpiego). Initially we planned to recruit pregnant women into G-ANC as they came to health posts for routine ANC. However, insufficient numbers of women were accessed at the health posts for ANC. This led to a change in the recruitment strategy where pregnant women were identified through the following four methods: (1) recruitment by midwives at healthcare centers, (2) outreach at health posts by HEWs, (3) home visits by HEWs, and (4) engagement through existing community structures such as the Women’s Development Army (WDA) [33]. The WDA is a structured network of volunteer women that provide health promotion services and facilitate communication between households and HEW in their respective communities [34]. The gestational age of prospective participants was estimated by trained HEWs using maternal history (e.g., last menstrual period, perception of fetal movement or quickening) and gestational age estimation based on fundal height measurement using a measuring tape. All eligible individuals who were willing to participate and provide consent were included in the pilot study. The pilot study aspired to enroll at least eight women per health post. If the pilot sample size was not met, we planned to refrain from proceeding to the main study.

### Data collection procedures and instruments

Data were collected 25–29 October 2021 by research assistants, all of whom had previous qualitative research experience. They received 1 day of training on the study overview, procedures, and ethics before conducting the interviews. They were supervised by an experienced research associate. A total of 20 interviews, including 13 in-depth and 7 key informant interviews, were conducted with the following participants: five health extension workers (HEWs) who facilitated the two G-ANC meetings, one from each facility (total: 5); two pregnant women from each facility who completed the two G-ANC meetings and were available and willing to participate at the time of data collection (total: 10); three pregnant women who were identified through the attendance register as having attended the first G-ANC session but did not the second meeting, and were approached to explore barriers to retention; and two staff members from the implementing organization (Jhpiego) who were directly involved in G-ANC implementation. Aggregate participant characteristics are presented in Table 1. No additional interviews were conducted as thematic saturation was reached.

**Table 1:** Attendance of eligible pregnant women at G-ANC meetings by health post

**Health post (HP)**	**Number of eligible pregnant women identified**	**Total number of pregnant women at the start G-ANC meetings**	**Total number of pregnant women who attended Meeting 2**		**Average age of the women(in years)**	**Average gestational age (in weeks)**
			**Number**	**%**		
**HP1**	13	10	10	100	25.3	16.5
**HP2**	10	10	9	90	25.8	15
**HP3**	11	11	9	82	27.8	16.3
**HP4**	13	13	11	85	28.4	16.3
**HP5**	11	10	10	100	28.5	17.3
**Total**	58	54	49	91	27.14	16.4 ± (2.4)

*HP *health post, *G-ANC *group antenatal care. Percentage reflects the proportion of women who attended Meeting 2 out of those who started Meeting 1. Total average gestational age is reported as mean ± standard deviation (SD). Age is reported in years

Face-to-face interviews were conducted in a place convenient for the participants and in a manner that provided privacy. No repeat interviews were conducted. Interviews were conducted utilizing guides designed to explore and gather insights on availability of enough eligible women to form a group, the willingness of the women to attend future G-ANC meetings and perceptions on the ability of the HEWs in facilitating the meetings from participants. Interview notes for immediate recording of key insights, participant reactions, and contextual observations were utilized. All the interviews and discussions were audio recorded with the consent of the participant. Daily debriefing sessions were conducted with research assistants and associates to address emerging methodological and substantive issues. Data collection was conducted in accordance with COVID-19 protocols.

### Data analysis

Data analysis for the pilot study was conducted based on transcribed and translated interviews collected by the research assistants. Following each audio recording, either the moderator or an official transcriber familiar with local language transcribed audio recordings verbatim and translated it from Amharic to English. Open code qualitative data analysis software [35] was used to assist the data sorting, categorizing, and coding. Both predefined and emerging themes were used. This thematic framework was systematically applied to all interview transcripts. The transcribed data were initially coded based on a predefined codebook, and new codes were added as they emerged during analysis to capture common themes and key points. Two independent coders (SGA and WWY) conducted the coding for each transcript, and their codes were compared. Discrepancies were discussed and resolved through agreement. Identified themes were then discussed, summarized, and illustrated with relevant quotes for each topic.

## Results

**Fig. 1:**
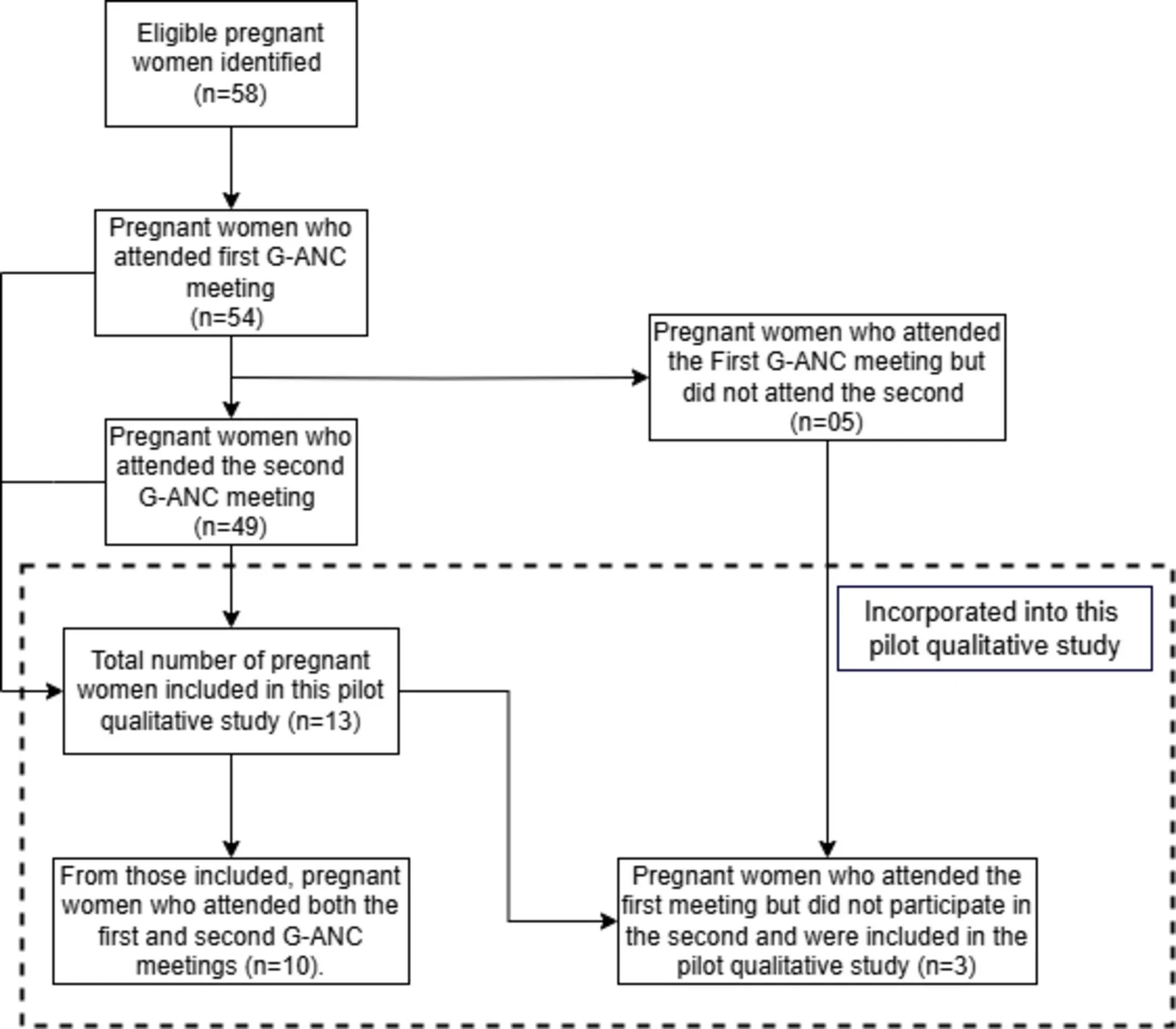
Participant selection and attendance flow diagram for the two G-ANC meetings

This pilot qualitative study included a total of 20 interviews: 13 in-depth interviews with pregnant women, 5 key informant interviews with HEWs trained to facilitate G-ANC meetings (one from each facility), and 2 field staff from Jhpiego who introduced the intervention. Initially, 58 eligible pregnant women were identified, of whom 54 participated in the first G-ANC meeting. Among these, 49 attended the second meeting, while 5 did not. The qualitative sample of pregnant women consisted of 13 participants: 10 who attended both meetings and 3 who participated in the first meeting but were absent for the second (Fig. 1).

### Background characteristics of participants in the study

The participant pregnant women ages ranged from 20 to 39 years. All were married, and all of the pregnant women were from rural residences. The average age of the women was 27.1 and the average gestational age at enrolment into the G-ANC meetings was 16.4 weeks (± 2.4) (Table 1).

Study findings are organized into three key thematic areas assessing feasibility: (1) HEW perceptions around availability of adequate numbers of eligible pregnant women to form G-ANC groups, (2) pregnant women’s willingness to be enrolled and attend successive future G-ANC meetings, and (3) ability of HEWs to facilitate the G-ANC meetings.

### Theme 1: HEW perceptions around availability of adequate numbers of eligible pregnant women to form G-ANC groups

All health posts were successful at enrolling at least eight women, identified early in pregnancy, to form a G-ANC group (Table 1). HEWs utilized various techniques to ensure that the necessary number of pregnant women were identified and enrolled to attend the first meeting. One HEW stated,

Yes, it is possible to recruit the required number of women to start the meetings. We employed various strategies to enroll pregnant women with similar gestational ages, including house-to-house visits, asking community members about suspected pregnancies, engaging the Women’s Development Army, visiting areas where women gather, such as water collection points, identifying and approaching husbands whose wives might be pregnant, and identifying women who missed funeral ceremonies in the community.

Although most HEWs indicated there were enough eligible women, some reported challenges in identifying and locating eligible women for G-ANC groups. Women were reluctant to disclose their pregnancy, lacked knowledge of their last menstrual period to estimate gestational age, and were hesitant to seek health services unless facing severe health issues. Additionally, HEWs highlighted time constraints as a recruitment challenge. One HEW remarked, “Yes, we identified more than enough women with the same gestational age in a short period, that is 5 days. For the future, it would be good to have 2–3 weeks so that we can get more. For example, in XX site, we did not incorporate women living far away, but if we had more time, we could have visited their homes and enrolled them.”

Developing close relationships by the HEWs within the communities and especially with the pregnant women was crucial in overcoming such recruitment challenges. One HEW stated, “The challenge is that, culturally, pregnant women often prefer not to disclose their pregnancy during the first few months. However, since we built a close relationship with them and earned their trust, they felt comfortable sharing their pregnancy with us, allowing us to include them in the G-ANC meetings.” Furthermore, utilizing local community and village structures such as the WDA was beneficial for enrollment. One HEW expressed the experience by stating, "We can now get these women using existing community structures through the women’s development army."

### Theme 2: Pregnant women’s willingness to enroll and participate in successive future G-ANC meetings

Most women were willing to enroll and continue participation in G-ANC meetings due to the convenience of accessing ANC services within their village at the health post (as opposed to traveling farther to the health center), the ability to measure their own blood pressure and weight, the opportunity to engage in peer discussions, and the chance to pose questions and get answers during G-ANC meetings. Participants also highlighted the potential benefits of developing skills in public speaking, enhancing self-confidence, and fostering decision-making abilities regarding their health.

One pregnant woman who attended both G-ANC meetings explained, “Before attending this group discussion, I used to lift heavy materials or household equipment without care for my pregnancy. However, after participating in the discussion, I started taking better care of myself. I now take two-hour naps to ensure I get enough rest, I avoid lifting heavy weights, eat nutritious foods for my health and fetus’s health, and monitor for any danger signs during my pregnancy. If I notice any danger signs, I make sure to visit the health facility promptly.”

Among women who did not attend the second G-ANC meeting, barriers to participation-included workloads associated with household chores, illness, and meetings not adhering to schedules, primarily due to waiting to start until all the women have arrived. One women who attended the first session but did not return for the second indicated, “One challenge was that not everyone was punctual for the scheduled meetings. Some of us would arrive between 8:00 and 9:00 in the morning, while others showed up around midday.”

Despite these challenges, most women expressed interest in attending future G-ANC meetings. One woman, who missed the second meeting due to personal reasons but was interested to attend future meetings stated that, “I have attended one group antenatal session, but I was absent in the second session because my sister was sick. I will be rejoining the group for the next session.”

### Theme 3: Ability of HEW to facilitate G-ANC meetings

HEWs were able to lead and facilitate the G-ANC meetings successfully. Participants unanimously acknowledged the capability of HEWs in facilitating and leading G-ANC meetings. The HEWs adhered to the meeting schedule and content. HEW attributed this success to the training they received on recruitment, enrollment, meeting facilitation, and tracking attendance. One Jhpiego staff member with previous on-site intervention experience conducting G-ANC at the health center level indicated that the performance of the HEWs was comparable to that of the midwives they had supervised. They stated, “Yes, the meeting was conducted very well. I have previous experience with G-ANC at the health center level, and I found the facilitation to be excellent. The way the meeting was handled was impressive because I have seen G-ANC facilitated by midwives in the ENAT project. With these HEWs, the session was interactive, and the women were happy.”

While the HEWs had equivalent educational backgrounds, some variation in facilitation skills was identified during the training. During the actual meetings, close supportive supervision by on-site intervention staff allowed for the identification of staff needing support and the provision of feedback. Another Jhpiego on-site intervention staff member further indicated,

In XX, we have three sites, and during the evaluation of the facilitators at each site, we identified gaps among some G-ANC facilitators during the training. Specifically, one site had challenges primarily with clinical-related tasks after the training. To address this, we prioritized support for that site. I was present at the site before the first meeting began, allowing me to observe the process from start to finish and assist with clinical-related tasks. After the meeting, we provided feedback to the facilitators. Comparing the first and second meetings, I observed significant improvements; the facilitators performed much better during the second meeting. I believe this process successfully addressed the identified gaps.

Facilitators found the meeting guide helpful to guide each meeting and improve facilitation. One HEW who facilitated the meetings indicated, “The meeting guide helps me communicate effectively with the pregnant women, making it easier to facilitate the discussion. Our communication with the women has become so strong that it feels like a family bond. This creates a comfortable environment where they feel free to openly share their emotions during pregnancy and ask any questions they may have.”

## Discussion

The findings from our pilot study suggest that implementing G-ANC at the health post level is feasible in the Amhara region, Ethiopia. An adequate number of eligible pregnant women were available at all health posts to form G-ANC groups, and almost all pregnant women attended G-ANC meetings, with most participants affirming their willingness to participate in successive future G-ANC meetings. HEWs effectively facilitated the G-ANC meetings, demonstrating a strong understanding of the G-ANC model. This pilot study suggests that future implementation of the complete G-ANC model (i.e., all six meetings) at the health post level is feasible.

Our findings indicate that a sufficient number of eligible pregnant women were successfully identified to form G-ANC groups, aligning with results from other feasibility studies [17, 30, 32, 33, 36]. HEWs acknowledged that multiple strategies were needed to identify enough eligible women. Some noted difficulties in identifying and locating eligible women early in pregnancy mainly due to pregnant women’s hesitancy to disclose pregnancy early on, lack of knowledge about their last menstrual period to be able to estimate gestational age, and hesitancy to seek health services unless experiencing severe health issues. Because women do not usually come for ANC early in pregnancy to the health post or health center, HEWs were able to overcome recruitment and enrollment challenges by leveraging existing community structures. The utilization of community level structures for recruitment and enrollment of pregnant women for ANC follow-up has been noted as an effective strategy in a study conducted in East Africa [37].

Most pregnant women showed a positive response and were willing to enroll and participate in G-ANC meetings. The primary reasons included the convenience of accessing ANC services within their village at the health post. Accessibility is one of the factors that affects health service utilization in ANC services [38]. Furthermore, they appreciated the opportunity to measure their own blood pressure and weight, engage in peer discussions, and pose questions. Pregnant women who participated in the study highlighted benefits such as enhancing self-confidence, and improving decision-making abilities regarding their health. This aligns with other studies on women’s experiences and perceptions of G-ANC [39, 40–43].

Participants expressed confidence in the trained HEWs in facilitating the meetings. Health posts are not typically equipped for ANC follow-up because they lack the necessary resources, such as laboratories. However, comprehensive health posts that provide POCT can bring quality ANC services close to the community, especially in rural areas. HEWs spend up to 75% of their time within communities and are primary sources of information for various maternal and reproductive health issues which makes them ideal facilitators for G-ANC meetings [21].

The results of this study, which informed the main study, suggest that improved support for HEWs in facilitating G-ANC meetings, along with post-training supervision and feedback, is crucial for addressing challenges in gestational age estimation and ensuring effective meeting facilitation. A comparison of the first and second meetings showed significant progress, highlighting the positive influence of the feedback mechanism. Furthermore, the implementation of a meeting guide was crucial in improving the structure and delivery of communication, creating a space where expectant mothers could freely express their experiences and concerns. Some HEWs needed additional support to perform ANC clinical tasks. Future G-ANC implementation research, including the main study, should apply a comprehensive approach incorporating HEWs, district health office, zonal health department and regional health office in strategic planning for successful implementation [44]. Learnings from this pilot study revealed several amendments for incorporation in the main study (Table 2).

**Table 2:** Summary of pilot learnings and main study amendments

Learnings	Amendments
Limited knowledge of HEWs on gestational age estimation	Integrate gestational age estimation skills into G-ANC facilitation skill training using classroom-based simulation exercises and practical attachments at selected health centers
Short recruitment time between HEW training and screening	Provide an additional week (combined with the previous 1 week there will be a total of 2 weeks) following community mobilization orientation for HEWs to screen and enroll women
Limited engagement of reproductive health officers at district and zonal level	Include reproductive health officers at district and zonal levels into G-ANC facilitation and mentorship skill training
Identified recurring or redundant discussion topics and messages across G-ANC meetings, leading to meetings being perceived as overly long or drawn out	Revise illustration cards and G-ANC meeting facilitation guide

*HEW *health extension worker, *G-ANC *group antenatal care

A key strength of this pilot research is its inclusion of diverse stakeholders (e.g., women with high and low retention, HEWs, and intervention implementers) to capture a wide range of perspectives, providing a balanced view of the intervention’s implementation, challenges, and impacts. The study is limited by its short observation period of two meetings, which does not represent the full G-ANC model of six meetings. While the findings support feasibility, expressed willingness to attend future meetings does not necessarily equate with actual follow-through, and attrition over the remaining four meetings remains unknown. The sample was purposively selected, and women who completed both meetings and were available at the time of data collection may have been more positively disposed toward G-ANC than non-attendees, potentially introducing selection bias. Individual-level demographic details for interview participants beyond what is reported in Table 1 (e.g., education, parity) were not collected, which limits the depth of participant characterization. Responses from implementer participants may be subject to social desirability bias, given their role as part of the implementation staff. Additionally, the study was conducted during the COVID-19 pandemic, and it is possible that the public health context influenced women's willingness to attend facility-based services in ways that may not reflect routine implementation conditions.

## Conclusion

The pilot study affirms the feasibility of introducing G-ANC at the health post level. Prior to the study, three specific feasibility concerns were anticipated: (1) whether an adequate number of eligible pregnant women of similar gestational age could be identified and enrolled; (2) whether pregnant women would be willing to enroll and participate in successive G-ANC meetings; and (3) whether HEWs would have sufficient capacity to facilitate the G-ANC meetings effectively. None of these major anticipated challenges were encountered. Existing community structures were instrumental in achieving adequate enrollment, and HEWs demonstrated sufficient capacity to facilitate G-ANC meetings with the support of structured training and meeting guides. The results of the pilot study informed the design of a cluster randomized trial to evaluate the acceptability and effectiveness of G-ANC at the health post level on ANC attendance and facility-based delivery.

## Supplementary Information

Supplementary Material 1: G-ANC CONSORT-Checklist.

## Data Availability

No quantitative data are associated with this manuscript. Tools used for data collection are available upon request.

## Abbreviations

ACIPH: Addis Continental Institute of Public Health
ANC: Antenatal care
CHW: Community health worker
EDHS: Ethiopia Demographic and Health Survey
HEW: Health extension workers
HP: Health post
IDI: In-depth interview
KII: Key informant interview
G-ANC: Group antenatal care
POCT: Point of care testing
WDA: Women development Army

## References

[1] World Health Organization. WHO recommendations on antenatal care for a positive pregnancy experience. Geneva: World Health Organization; 2016. p. 152. 28079998

[2] Atlaw D, Charkos TG, Kasim J, Chatu VK. Why does the number of antenatal care visits in Ethiopia remain low?: A Bayesian multilevel approach. PLoS ONE. 2024May 3;19(5):e0302560. 10.1371/journal.pone.0302560PubMedPMID:38701069;PubMedCentralPMCID:PMC11068190. 38701069 10.1371/journal.pone.0302560 PMC11068190

[3] Central Statistical Agency (CSA) [Ethiopia] and ICF. Ethiopia Demographic and Health Survey 2016. Addis Ababa, Ethiopia and Rockville, Maryland, USA: CSA and ICF; 2016.

[4] Central Statistical Agency [Ethiopia] and ICF International. Ethiopia Demographic and Health Survey 2011. Addis Ababa, Ethiopia and Calverton, Maryland, USA: Central Statistical Agency and ICF International; 2012.

[5] Central Statistical Agency [Ethiopia] and ORC Macro. Ethiopia Demographic and Health Survey 2005. Addis Ababa, Ethiopia and Calverton, Maryland, USA: Central Statistical Agency and ORC Macro; 2006.

[6] Ethiopian Public Health Institute (EPHI) [Ethiopia] and ICF. Ethiopia Mini Demographic and Health Survey 2019: Final Report. Addis Ababa, Ethiopia, and Rockville, Maryland, USA: EPHI and ICF; 2021.

[7] Wake SK, Botore A, Mohammed A, Gemede K, Bariso M, Gerema U. Disparities in Antenatal Care Visits between Urban and Rural Ethiopian Women. J Pregnancy. 2023Sep;27(2023):9031344. 10.1155/2023/9031344PubMedPMID:37799709;PubMedCentralPMCID:PMC10550413. 10.1155/2023/9031344 PMC10550413 37799709

[8] Berhan Y, Berhan A. Antenatal Care as a Means of Increasing Birth in the Health Facility and Reducing Maternal Mortality: A Systematic Review. Ethiop J Health Sci. 2014Sep;12(24):93–104. 10.4314/ejhs.v24i0.9S. 10.4314/ejhs.v24i0.9s PMC4249212 25489186

[9] Drivers and deterrents of facility delivery in sub-Saharan Africa: a systematic review | Reproductive Health | Full Text. Cited 2024 Oct 15. Available from: https://reproductive-health-journal.biomedcentral.com/articles/10.1186/1742-4755-10-40. 10.1186/1742-4755-10-40 PMC3751820 23962135

[10] Mbuagbaw L, Medley N, Darzi AJ, Richardson M, Habiba Garga K. Health system and community level interventions for improving antenatal care coverage and health outcomes. Cochrane Database Syst Rev. 2015;2015(12):CD010994. 10.1002/14651858.CD010994.pub2. 26621223 10.1002/14651858.CD010994.pub2 PMC4676908

[11] Quality of antenatal care predicts retention in skilled birth attendance: a multilevel analysis of 28 African countries | BMC Pregnancy and Childbirth | Full Text. Cited 2024 Jul 6. Available from: https://bmcpregnancychildbirth.biomedcentral.com/articles/10.1186/s12884-017-1337-1 10.1186/s12884-017-1337-1 PMC5445515 28545422

[12] FMOH. Federal Minstry of Health, National Antenatal Care Guideline. Ensuring Positive Pregnancy Experience!. 2022. Available from: http://repository.iphce.org/xmului/handle/123456789/1647

[13] Gaur BPS, Vasudevan J, Pegu B. Group Antenatal Care: A Paradigm Shift to Explore for Positive Impacts in Resource-poor Settings. J Prev Med Public Health. 2021Jan 31;54(1):81–4. 10.3961/jpmph.20.349. 33618503 10.3961/jpmph.20.349 PMC7939754

[14] Kabue MM, Grenier L, Suhowatsky S, Oyetunji J, Ugwa E, Onguti B, et al. Group versus individual antenatal and first year postpartum care: study protocol for a multi-country cluster randomized controlled trial in Kenya and Nigeria. Gates Open Res. 2018;2:56. 10.12688/gatesopenres.12867.1. 30706056 10.12688/gatesopenres.12867.1 PMC6350506

[15] Ickovics JR, Kershaw TS, Westdahl C, Magriples U, Massey Z, Reynolds H, et al. Group prenatal care and perinatal outcomes: a randomized controlled trial. Obstet Gynecol. 2007;110(2):330–9. 10.1097/01.AOG.0000275284.24298.23. 17666608 10.1097/01.AOG.0000275284.24298.23 PMC2276878

[16] Ickovics JR, Reed E, Magriples U, Westdahl C, Schindler Rising S, Kershaw TS. Effects of group prenatal care on psychosocial risk in pregnancy: Results from a randomised controlled trial. Psychol Health. 2011Feb;26(2):235–50. 10.1080/08870446.2011.531577. 21318932 10.1080/08870446.2011.531577 PMC3311036

[17] Gareau S, Lòpez-De Fede A, Loudermilk BL, Cummings TH, Hardin JW, Picklesimer AH, et al. Group Prenatal Care Results in Medicaid Savings with Better Outcomes: A Propensity Score Analysis of CenteringPregnancy Participation in South Carolina. Matern Child Health J. 2016Jul;20(7):1384–93. 10.1007/s10995-016-1935-y. 26979611 10.1007/s10995-016-1935-y

[18] Picklesimer AH, Billings D, Hale N, Blackhurst D, Covington-Kolb S. The effect of CenteringPregnancy group prenatal care on preterm birth in a low-income population. Am J Obstet Gynecol. 2012May;206(5):415.e1-415.e7. 10.1016/j.ajog.2012.01.040. 22542115 10.1016/j.ajog.2012.01.040

[19] Manant A, Dodgson JE. CenteringPregnancy: An Integrative Literature Review. J Midwife Womens Health. 2011Mar;56(2):94–102. 10.1111/j.1542-2011.2010.00021.x. 10.1111/j.1542-2011.2010.00021.x 21429072

[20] Grenier L, Suhowatsky S, Kabue MM, Noguchi LM, Mohan D, Karnad SR, et al. Impact of group antenatal care (G-ANC) versus individual antenatal care (ANC) on quality of care, ANC attendance and facility-based delivery: A pragmatic cluster-randomized controlled trial in Kenya and Nigeria. Gutman J, editor. PLoS ONE. 2019 Oct 2;14(10):e0222177. 10.1371/journal.pone.0222177 10.1371/journal.pone.0222177 PMC6774470 31577797

[21] Patil CL, Abrams ET, Klima C, Kaponda CPN, Leshabari SC, Vonderheid SC, et al. CenteringPregnancy-Africa: A pilot of group antenatal care to address Millennium Development Goals. Midwifery. 2013Oct;29(10):1190–8. 10.1016/j.midw.2013.05.008. 23871278 10.1016/j.midw.2013.05.008 PMC3786019

[22] McKinnon B, Sall M, Vandermorris A, Traoré M, Lamesse-Diedhiou F, McLaughlin K, et al. Feasibility and preliminary effectiveness of group antenatal care in Senegalese health posts: a pilot implementation trial. Health Policy Plan. 2020Jun 1;35(5):587–99. 10.1093/heapol/czz178. 32155254 10.1093/heapol/czz178

[23] Lori JR, Ofosu-Darkwah H, Boyd CJ, Banerjee T, Adanu RMK. Improving health literacy through group antenatal care: a prospective cohort study. BMC Pregnancy Childbirth. 2017Jul 14;17(1):228. 10.1186/s12884-017-1414-5PubMedPMID:28705179;PubMedCentralPMCID:PMC5513199. 28705179 10.1186/s12884-017-1414-5 PMC5513199

[24] Agarwal A, Mann C, Abdella E, Mitiku W, Alebachew A, Berman P. Recurrent costs in primary health care in Ethiopia: facility and disease specific unit costs and their components in government primary hospitals and health centers. BMC Health Serv Res. 2020May 7;20(1):389. 10.1186/s12913-020-05218-1. 32381077 10.1186/s12913-020-05218-1 PMC7204209

[25] MOH. Realizing universal health coverage through primary healthcare. A roadmap for optimizing the Ethiopian health extension program 2020 - 2035. MOH; 2020.

[26] Demissie M, Abera N, Gebremeskel L, Tadesse D, Dadi TL, Zebere T, Tigabu S, Fentaye FW, Alemayehu YK, Teklu AM, Medhin G, Zeleke S. Ethiopian Health Extension Workers' Training Status and Perceived Competency. Ethiop J Health Sci. 2023;33(1 Suppl)–S60. 10.4314/ejhs.v33i1.6S. PMID: 38362476; PMCID: PMC10866291. 10.4314/ejhs.v33i1.6S PMC10866291 38362476

[27] Shibre G, Mekonnen W. Socio-economic inequalities in ANC attendance among mothers who gave birth in the past 12 months in Debre Brehan town and surrounding rural areas, North East Ethiopia: a community-based survey. Reprod Health. 2019Dec;16(1):99. 10.1186/s12978-019-0768-8. 31286965 10.1186/s12978-019-0768-8 PMC6615209

[28] Asefa A, Gebremedhin S, Messele T, Letamo Y, Shibru E, Alano A, et al. Mismatch between antenatal care attendance and institutional delivery in south Ethiopia: a multilevel analysis. BMJ Open. 2019;9(3):e024783. 10.1136/bmjopen-2018-024783. 30898814 10.1136/bmjopen-2018-024783 PMC6527994

[29] Federal Ministry of Health (FMOH). National Antenatal Care Guideline (Ensuring Positive Pregnancy Experience). 2022. Available from: https://www.moh.gov.et/sites/default/files/2024-07/National%20Antenatal%20Care%20Guideline_2022.pdf

[30] Lee AC, Abate FW, Mullany LC, Baye E, Berhane YY, Derebe MM, et al. Enhancing nutrition and antenatal infection treatment (ENAT) study: protocol of a pragmatic clinical effectiveness study to improve birth outcomes in Ethiopia. BMJ Paediatr Open. 2022;6(1):e001327. 10.1136/bmjpo-2021-001327. 10.1136/bmjpo-2021-001327 PMC8762145 36053580

[31] Lundeen T, Musange S, Azman H, Nzeyimana D, Murindahabi N, Butrick E, et al. Nurses’ and midwives’ experiences of providing group antenatal and postnatal care at 18 health centers in Rwanda: a mixed methods study. PLoS ONE. 2019;14(7):e0219471. 10.1371/journal.pone.0219471. 31295335 10.1371/journal.pone.0219471 PMC6622527

[32] Effect of the enhancing nutrition and antenatal infection treatment (ENAT) intervention on birth weight in Ethiopia: a cluster randomized controlled trial - PubMed. Cited 2024 Mar 7. Available from: https://pubmed.ncbi.nlm.nih.gov/37644454/10.1186/s12884-023-05912-y PMC10466862 37644454

[33] Yallew WW, Fasil R, Berhanu D, Wolde K, Teshite D, Sethi R, et al. Evaluation of the feasibility, acceptability, and impact of group antenatal care at the health post level on continuation in antenatal care and facility based delivery in Ethiopia using a cluster randomized stepped-wedge design: study protocol. Gates Open Res. 2024. 10.12688/gatesopenres.15190.1. 39936142 10.12688/gatesopenres.15190.3 PMC11813170

[34] Maes K, Closser S, Tesfaye Y, Gilbert Y, Abesha R. Volunteers in Ethiopia’s women’s development army are more deprived and distressed than their neighbors: cross-sectional survey data from rural Ethiopia. BMC Public Health. 2018Feb 14;18(1):258. 10.1186/s12889-018-5159-5. 29444660 10.1186/s12889-018-5159-5 PMC5813408

[35] Open Code. Cited 2024 Jun 2. Available from: https://www.umu.se/en/department-of-epidemiology-and-global-health/research/open-code2/

[36] Sawtell M, Wiggins M, Wiseman O, Mehay A, McCourt C, Sweeney L, et al. Group antenatal care: findings from a pilot randomised controlled trial of REACH Pregnancy Circles. Pilot and Feasibility Studies. 2023;9(1):42. 10.1186/s40814-023-01238-w. 36927579 10.1186/s40814-023-01238-w PMC10018939

[37] Wafula ST, Nalugya A, Kananura RM, Mugambe RK, Kyangwa M, Isunju JB, et al. Effect of community-level intervention on antenatal care attendance: a quasi-experimental study among postpartum women in Eastern Uganda. Glob Health Action. 2022Dec 31;15(1):2141312. 10.1080/16549716.2022.2141312. 36369730 10.1080/16549716.2022.2141312 PMC9665095

[38] Alibhai KM, Ziegler BR, Meddings L, Batung E, Luginaah I. Factors impacting antenatal care utilization: a systematic review of 37 fragile and conflict-affected situations. Confl Heal. 2022Jun 11;16(1):33. 10.1186/s13031-022-00459-9. 10.1186/s13031-022-00459-9 PMC9188725 35690840

[39] Patil CL, Klima CS, Leshabari SC, Steffen AD, Pauls H, McGown M, et al. Randomized controlled pilot of a group antenatal care model and the sociodemographic factors associated with pregnancy-related empowerment in sub-Saharan Africa. BMC Pregnancy Childbirth. 2017;17(2):336, 336. 10.1186/s12884-017-1493-3. 10.1186/s12884-017-1493-3 PMC5688418 29143624

[40] Hunter LJ, Da Motta G, McCourt C, Wiseman O, Rayment JL, Haora P, et al. Better together: A qualitative exploration of women’s perceptions and experiences of group antenatal care. Women and Birth. 2019Aug 1;32(4):336–45. 10.1016/j.wombi.2018.09.001. 30253938 10.1016/j.wombi.2018.09.001

[41] Jolivet RR, Uttekar BV, O’Connor M, Lakhwani K, Sharma J, Wegner MN. Exploring perceptions of group antenatal Care in Urban India: results of a feasibility study. Reprod Health. 2018Dec;15(1):57. 10.1186/s12978-018-0498-3. 29615069 10.1186/s12978-018-0498-3 PMC5883286

[42] Ibañez-Cuevas M, Heredia-Pi IB, Fuentes-Rivera E, Andrade-Romo Z, Alcalde-Rabanal J, Cacho LBB, et al. Atención Prenatal en Grupo en México: perspectivas y experiencias del personal de salud. Rev saúde pública. 2020Dec;12(54):140. 10.11606/s1518-8787.2020054002175.

[43] Group versus conventional antenatal care for women - PMC. Cited 2023 Dec 22. Available from: https://www.ncbi.nlm.nih.gov/pmc/articles/PMC6465187/

[44] Pekkala J, Cross-Barnet C, Kirkegaard M, Silow-Carroll S, Courtot B, Hill I. Key considerations for implementing group prenatal care: lessons from 60 practices. J Midwifery Womens Health. 2020;65(2):208–15. 10.1111/jmwh.13047. 31642589 10.1111/jmwh.13047

